# A novel risk-scoring system conducing to chemotherapy decision for patients with pancreatic ductal adenocarcinoma after pancreatectomy

**DOI:** 10.7150/jca.57768

**Published:** 2021-05-27

**Authors:** Yuqiang Li, Mengxiang Tian, Yuan Zhou, Fengbo Tan, Wenxue Liu, Lilan Zhao, Daniel Perez, Xiangping Song, Dan Wang, Christine Nitschke, Qian Pei, Cenap Güngör

**Affiliations:** 1 Department of General Surgery, Xiangya Hospital, Central South University, Changsha, China.; 2 Department of General Visceral and Thoracic Surgery, University Medical Center Hamburg-Eppendorf, Hamburg, Germany.; 3 Department of Cardiology, Xiangya Hospital, Central South University, Changsha, China.; 4 Department of Rheumatology, Guangdong Provincial People's Hospital, Guangdong Academy of Medical Sciences, Guangzhou, China.; 5 Department of Thoracic Surgery, Fujian Provincial Hospital, Fuzhou, China.

**Keywords:** PDAC, nomogram, chemotherapy, SEER database, surgical resection

## Abstract

**Background:** Chemotherapy is suggested to use in all stages of pancreatic cancer. Is it reasonable to recommend chemotherapy for all PDAC patients? It is necessary to distinguish low-risk PDAC patients underwent pancreatectomy, who may not lose survival time due to missed chemotherapy and not need to endure pain, nausea, tiredness, drowsiness, and breath shortness caused by chemotherapy.

**Methods:** Nomograms were constructed with basis from the multivariate Cox regression analysis. X-tile software was utilized to perform risk stratification. Survival curves were used to display the effect of chemotherapy in different risk-stratification.

**Results:** All of the significant variables were used to create the nomograms for overall survival (OS). The total risk score of each patient was calculated by summing the scores related to each variable. X-tile software was utilized to classify patients into high-risk (score >283), median-risk (197<score ≤283), and low-risk (score ≤197) according to the total risk score. The low-risk PDAC patients after pancreatectomy cannot gain survival benefit from chemotherapy after surgery (p=0.443). Moreover, chemotherapy improved survival for patients with resected PDAC in the median-risk (p<0.001) and high-risk (p<0.001) groups.

**Conclusions:** our research constructed a new risk-scoring system based on survival nomogram to screen low-risk PDAC patients after pancreatectomy and confirmed that those can avoid enduring side effects caused by chemotherapy without affecting the survival time.

## Introduction

Pancreatic adenocarcinoma retains the worst prognosis among all gastrointestinal malignancies with growing steadily incidence in the last two decades 1. Pancreatic ductal adenocarcinoma (PDAC) is the main histological type of pancreatic tumors and accounts for about 85% of cases 2, 3. Multiple factors are responsible for the poor prognosis, including early metastatic locoregional, unusual aggressiveness, the lack of effective systemic therapies and distant spread of pancreatic cancer cells 4.

Chemotherapy is suggested, by the National Comprehensive Cancer Network (NCCN) guidelines and the European Society for Medical Oncology-European Society of Digestive Oncology (ESMO-ESDO) guidelines, to use in all stages of pancreatic cancer 5, 6. However, is it reasonable to recommend chemotherapy for all PDAC patients? A meta-analysis including five randomized controlled trials showed that adjuvant chemotherapy only provided an extra 3 months of median survival time for patients with resected PDAC 7. A recent study confirmed that chemotherapy even cannot provide survival benefit for patients with early-stage PDAC 8. Moreover, experiences from the treatment of other tumors, such as colorectal cancer, gastric cancer, are able to affirm that early-stage tumors can be completely cured by surgical resection. Therefore, it is necessary to distinguish low-risk PDAC patients after pancreatectomy, who may not lose survival time due to missed chemotherapy and not need to endure pain, nausea, tiredness, drowsiness, and breath shortness caused by chemotherapy.

An effective risk scoring system is crucial to screen low-risk PDAC patients after pancreatectomy. Survival nomogram is a two-dimensional diagram giving a computation of mathematical functions and can calculate the risk score of independent prognostic factors based on survival time. Hence, the purpose of this study is to construct an effective scoring system based on survival nomogram stratifying the risk of patients with resected PDAC, and compare the effect of chemotherapy on PDAC patients after surgery at different risk levels.

## Materials and Methods

### Patients

**Figure 1:**
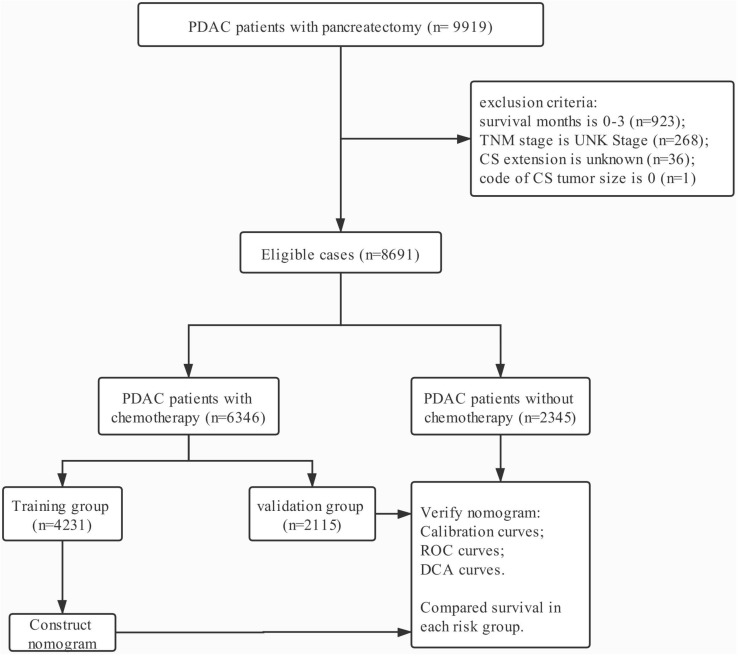
The flow chart.

Data in this retrospective analysis were extracted from the SEER linked database. The target population was limited to the patients with resected PDAC (ICD-O-3: 8140, 8141, 8144, 8160, 8170, 8210, 8211, 8255, 8260, 8261, 8263, 8290, 8310, 8323, 8342, 8350, 8430; RX Summ--Surg Prim Site (1998+): 10-90) diagnosed in the periods of 2004-2015, 9,919 patients in total. The exclusion criteria: survival months is 0-3 (n=923); TNM stage is UNK Stage (n=268); CS extension is unknown (n=36); code of CS tumor size is 0 (n=1). The final study sample contained 8,691 patients (Fig. 1). All procedures performed in this study were in line with the STROCSS criteria 9.

For each patient, the following data was acquired: age at diagnosis, gender, race, tumor size, tumor location, grade, T stage, N stage, M stage, regional nodes examined (RNE), extension, radiotherapy and chemotherapy. The final sample included 6,346 PDAC patients with chemotherapy and 2,345 ones without chemotherapy. PDAC patients with chemotherapy were used to build survival nomogram due to who received standard treatment as the guidelines, and then inconsistently separated into two groups (training group, n = 4231 and validation group, n = 2115). According to the eighth edition AJCC staging criteria, T staging was re-performed based on the tumor size (T1a-b: ≤1 cm; T1c: 1-2 cm T2: 2-4 cm; T3: >4 cm) and N staging was also re-classified based on the number of positive lymph nodes (N0: 0 positive regional lymph nodes; N1: one to three positive regional lymph nodes; N2: four or more positive regional lymph nodes). According to the code of CS extension, this study classified patients who were equivalent to the T1-2 staging in the seventh edition of AJCC as localized tumor, and those who matched with T3 staging as extrapancreatic extension.

### Statistical Analysis

An odds ratio (OR) and a 95% confidence interval (CI) were evaluated by univariable and multivariate Cox regression model. Variables with significant differences in univariate analysis were included in the Cox regression model for multivariate analysis. With the multivariate analysis results as the basis, by means of R 3.6.1 software (Institute for Statistics and Mathematics, Vienna, Austria; http://www.r-project.org/), nomograms were constructed. The prognostic prediction nomograms were validated by time-dependent receiver operating characteristics (ROC), decision curve analysis (DCA) and calibration curves. Statistical analyses were performed with IBM SPSS statistics trial ver. 25.0 (IBM, Armonk, NY, USA). All reported p-values lower than 0.05 were considered significant.

## Results

### Patient Characteristics

The characteristics of patients with resected PDAC enrolled from the SEER database are summarized in Table 1. The cohort is predominantly elderly patients (>60-year-old, 69.08%) with pancreatic head cancer (73.93%) in this study. Most of resected tumors are between 2 and 4 cm in size (T2, 53.85%). Moreover, metastatic lymph nodes were detected in 5,095 (58.62%) patients. Meanwhile, this study displayed extrapancreatic extension in 81.50% of patients. Patients receiving chemotherapy reach 73.02% of the entire cohort. Nevertheless, PDAC patients with limited extension and N0 stage incline to give up chemotherapy.

### Calibration and Verification of Prognostic Nomograms

**Figure 2:**
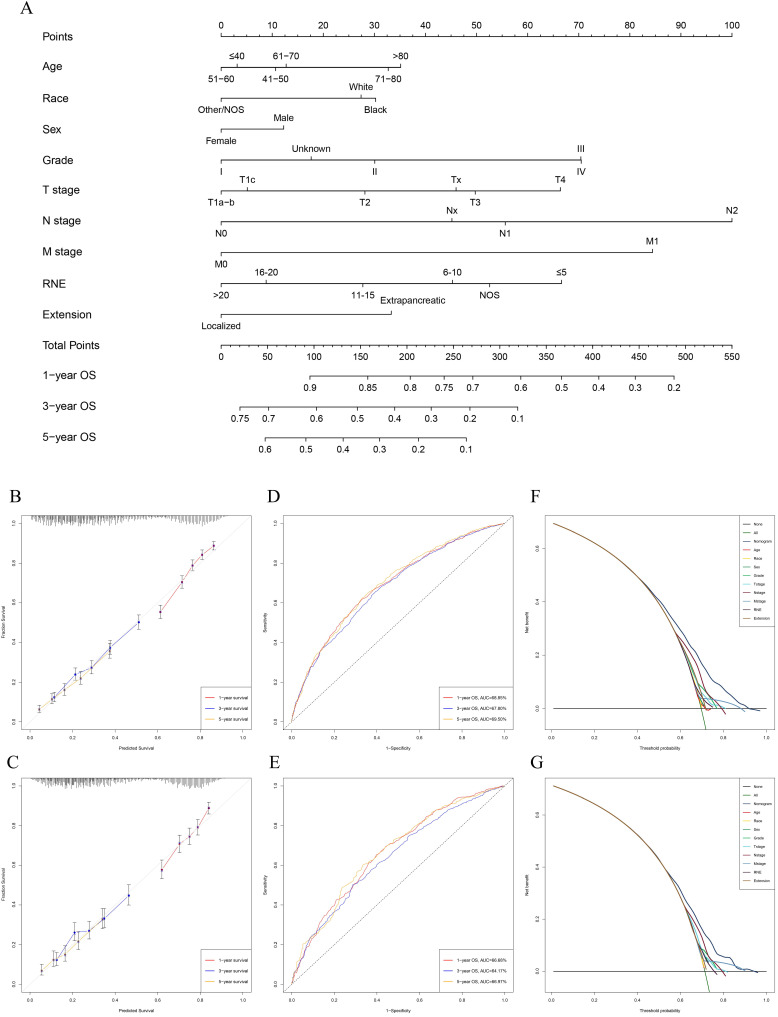
Construction and verification of the nomogram. **A:** The nomogram predicting OS for resectable PDAC patients with chemotherapy. **B:** The calibration curves predicting OS at 1-year, 3-year, 5-year in training group. **C:** The calibration curves predicting OS at 1-year, 3-year, 5-year in verification group. **D:** The AUC values of time- dependent ROC curves regarding nomogram predicting 1-year, 3-year, 5-year OS in training group. **E:** The AUC values of time- dependent ROC curves regarding nomogram predicting 1-year, 3-year, 5-year OS in verification group. **F:** The decision curve analysis displayed the obvious advantages of the nomogram comparing with the other indicators in training group. G: The decision curve analysis displayed the obvious advantages of the nomogram comparing with the other indicators in verification group.

**Figure 3:**
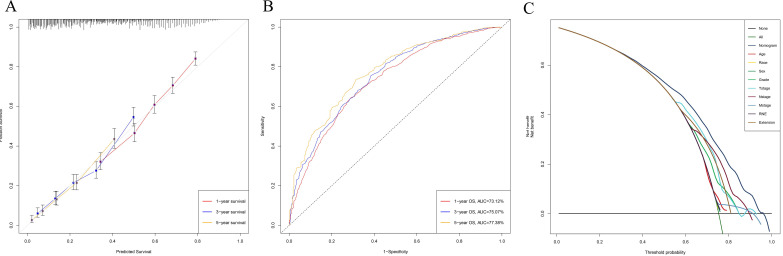
The calibration curve **(A)**, time-dependent ROC curve** (B)**, and DCA curve **(C)** showed favorable effects in resectable PDAC patients without chemotherapy.

Univariable and multivariable Cox regression analyses were used to calculate the effect of variables on overall survival (OS). The measure of the effect of each variable on OS was presented as the odds ratio (OR) and used to identify independent risk factors. Univariate analysis of variables with significant differences were included in the Cox regression model for multivariate analysis. According to the results based on the multivariate Cox regression analysis, OS is significantly associated with 9 variables, namely, age, race, sex, pathological grade, T stage, N stage, M stage, RNE and extension (Table 2). All of the significant variables were used to create the nomograms for OS. The prognostic nomogram for 1-, 3-, and 5-year OS is shown in Fig. 2A. The risk score of each independent prognostic factor is listed in Table 3. Various methods, including C-index value, time-dependent ROC curves, DCA curves and calibration curves, then were used to identify the discriminating superiority of nomogram. There are no obviously deviations from the reference line in calibration curves for OS in both training group and verification group (Fig. 2B-C), which demonstrating a high degree of reliability. Time-dependent receiver operating characteristics (ROC) at 1, 3, and 5 years are conducted to confirm that the nomogram have a favorable sensitivity and specificity (Fig. 2D-E). The DCA of the nomogram own excellent net benefits and is superior to the any single prognostic factors across the wider range of reasonable threshold probabilities, indicating outstanding value of clinical application (Fig. 2F-G). Moreover, the C-index values are 0.635 (95%CI: 0.624-0.646) in training cohort and 0.618 (95%CI: 0.603-0.633) in verification cohort respectively. Interestingly, the C-index value (0.658, 95%CI: 0.643-0.673), the time-dependent ROC curve, calibration curve, and decision curve also show favorable effects in resectable PDAC patients without chemotherapy (Fig. 3), verifying that this nomogram is also applicable to those who do not receive chemotherapy.

### Performance of the Nomograms in Stratifying on the basis of Risk Scores

**Figure 4:**
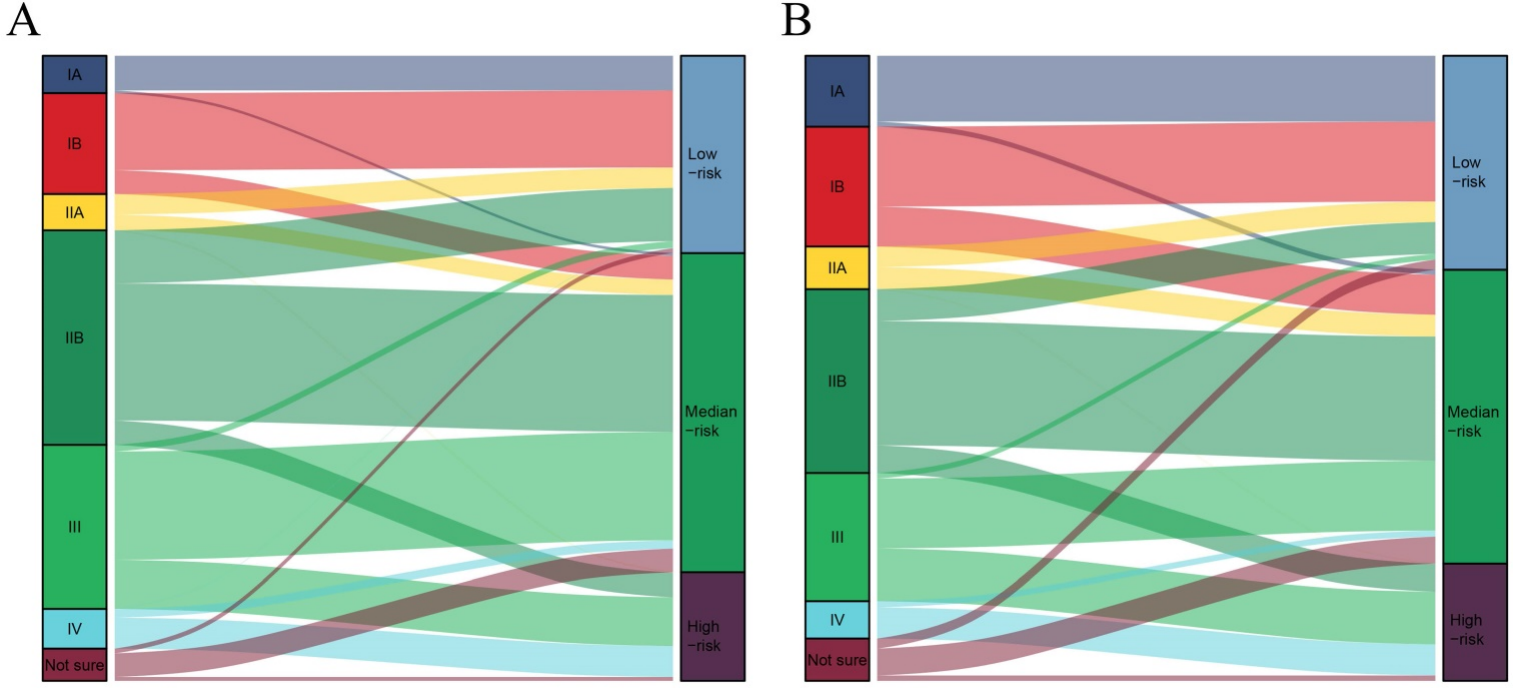
The correspondence between our risk stratification and the AJCC staging. **A:** The correspondence between our risk stratification and the AJCC staging in PDAC patients with chemotherapy. **B:** The correspondence between our risk stratification and the AJCC staging in PDAC patients without chemotherapy.

The total risk score of each patient was calculated by summing the scores related to each variable. X-tile software was utilized to classify patients into high-risk (score >283), median-risk (197<score ≤283), and low-risk (score ≤197) according to the total risk score (Supplementary Figure 1). Sankey diagrams were then delineated to show the correspondence between our risk stratification and the AJCC staging (Fig. 4). Patients in the low-risk group mainly originated from early-stage PDAC patients. However, the risk stratification cannot completely correspond to the AJCC staging system.

### The impact of chemotherapy on patients at each risk level

**Figure 5:**
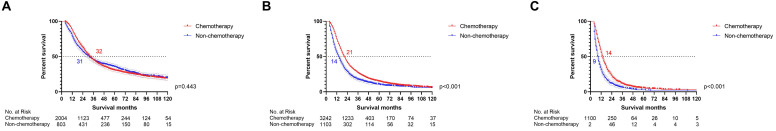
The survival differences between chemotherapy and non-chemotherapy in each risk stratification. **A:** The survival differences between chemotherapy and non-chemotherapy in PDAC patients with low-risk (p=0.443). **B:** The survival differences between chemotherapy and non-chemotherapy in PDAC patients with median-risk (p<0.001). **C:** The survival differences between chemotherapy and non-chemotherapy in PDAC patients with high-risk (p<0.001).

Can resectable PDAC patients at all risk levels really get survival benefit from chemotherapy? The survival differences between chemotherapy and non-chemotherapy patients were compared in each risk group by log-rank test respectively (Fig. 5). The survival curves indicated that the low-risk resectable PDAC patients cannot receive survival benefit from chemotherapy (p=0.443). Moreover, chemotherapy improved survival for resectable PDAC patients in the median-risk (p<0.001) and high-risk (p<0.001) groups. Therefore, our study successfully screened out low-risk resectable PDAC patients, who can avoid enduring side effects caused by chemotherapy without affecting the survival time.

## Discussion

As described in the guidelines, clinicians usually recommend chemotherapy for patients with PDAC to prolong survival since the unusual aggressiveness. Promising chemotherapy regimens, such as FOLFIRINOX, indeed demonstrated superiority 10. However, multi-drug chemotherapy leads to a highly toxic combination and serious adverse effects. Moreover, a recent study displayed that advances in surgery, including accurate assessment of the resection margins and total mesopancreatic excision (TMpE), contributed to the apparent improvement of survival in resectable PDAC patients, but progress in radiotherapy and chemotherapy was tedious for PDAC 11. Advanced surgical concepts may allow more resectable PDAC patients to avoid multi-drug chemotherapy or even chemotherapy. Moreover, a recent study reported that PDAC patients with stage IA cannot receive better survival from chemotherapy 8. These evidences motivated us to identify low-risk PDAC patients after pancreatectomy who do not need chemotherapy. To the best of our knowledge, our study was the first to look into a scoring system to screen out low-risk PDAC patients after pancreatectomy, who cannot obtain survival benefit from chemotherapy, based on public database. The successful scoring system also confirmed that chemotherapy for all PDAC patients is unreasonable.

Most of the studies usually focus on exploring optimal chemotherapy regimen and enroll more relatively advanced PDAC patients to investigate the effect of chemotherapy on survival. These studies, including more than 50% stage III PDAC patients 12, 13 or even over 70% ones with lymph nodes metastasis 14, 15, confirmed the chemotherapy superiority but ignored the fact that a small number of patients may not need chemotherapy. In addition, the guidelines do not formulate a corresponding chemotherapy regimen based on the pathological characteristics of resectable PDAC. Meanwhile, scarce research is not enough to support the rationality of chemotherapy for early-stage PDAC after resection. Therefore, these concepts involving chemotherapy for all PDAC patients need to be changed. A recent study reported that adjuvant chemotherapy was not associated with improved OS in PDAC patients with negative lymph nodes who underwent resection of pancreatic cancer after neoadjuvant chemotherapy 16, which indicates that some limited PDAC, that may be controlled by preoperative chemotherapy, can be completely cured by surgery without adjuvant chemotherapy. Another study further confirmed that chemotherapy cannot improve the survival of resectable PDAC patients with negative resectable lymph nodes by using a national database 17. However, it is not enough to judge whether chemotherapy is needed only by lymph node status, or even the AJCC staging system for resectable PDAC.

Previous studies reported the updated AJCC staging system owning better clinical value 18, 19. The nomogram displayed that N2 and T3 hold a higher risk score than N1 and extrapancreatic extension respectively, which can, to a certain extent, prove the superiority of the 8^th^ edition of AJCC staging compared to the 7^th^, at least for resectable PDAC. Nevertheless, the 8^th^ edition AJCC staging system is still not comprehensive enough for identifying low-risk groups. In fact, the predictive effect of the AJCC staging system, detected by C-index (nomogram: 0.637, 95%CI: 0.630-0.645; vs. the AJCC staging system: 0.616, 95%CI: 0.608-0.624), calibration curves, ROC curves and DCA curves, was not as good as the nomogram in this study (Supplementary Figure 2). In fact, the results regarding the survival difference between chemotherapy and non-chemotherapy in each AJCC stage display that only stage IA PDAC cannot obtain survival benefit from chemotherapy (Supplementary Figure 3), which matches the previous study 8. Furthermore, the early-stage PDAC patients defined by the AJCC staging system may be classified as intermediate or high-risk groups, as displayed by the Sankey diagrams. Therefore, it is necessary to incorporate other independent prognostic factors into the evaluation system. Moreover, the 8^th^ edition of AJCC staging of PDAC only considered the tumor size regardless of extrapancreatic extension for T3 5. Our study believed that AJCC staging should not completely ignore the extension since it is significantly related to OS. RNE is considered as the priority for the assessment of the quality of surgery 20, 21 and can serve as an independent prognostic factor for resectable PDAC patients. Increasing RNE is able to decrease the risk score for resectable PDAC in the nomogram. In fact, RNE is one of the key factors in determining whether a colorectal cancer patient needs chemotherapy 22. Similarly, RNE as an independent prognostic factor should be included in the chemotherapy decision-making system for PDAC after resection. Aggregately, the comprehensive scoring system can better serve for chemotherapy decisions-making than the AJCC staging system.

Plenty of studies did not provide detailed chemotherapy regimens to explore the effect of chemotherapy on survival 17, 23, 24, which may be influenced by that the guidelines never draft discriminate chemotherapy regimens for resectable PDAC patients with different risk, which weakens the defect in this study that we cannot get detailed chemotherapy regimens from the SEER database. Moreover, the data from the National Cancer Data Base (NCDB) demonstrated that T1/T2 PDAC patients have similar survival irrespective of the timing of chemotherapy and surgery and upfront resection is able to increase the possibility of long-term survival 23, which further supports our point that resection could completely cure some early-stage PDAC patients. Hence, despite the lack of detailed chemotherapy regimens and the timing of chemotherapy and surgery, the conclusion that the low-risk PDAC patients cannot obtain survival benefit from chemotherapy after resection is trustworthy. There were, unfortunately, a large number of patients with median- and high-risk in the non-chemotherapy group, who should obtain survival benefit from chemotherapy. Therefore, our risk scoring system can also be used to encourage these patients to actively receive chemotherapy.

The role of radiotherapy and indications for its use in this setting have been debated for some time and are still under investigation. Some studies indicated that radiotherapy can improve marginal negative resection and local control of PDAC 25. However, some scholars believed that the use of radiotherapy in pancreatic cancer has ended due to the poor results of several important radiotherapy trials 26. PDAC, being surrounded by many radiosensitive organs but with extremely treatment-resistant, actually bears high risks and low benefits in the case of receiving radiotherapy, which also cannot be used as an independent prognostic factor in this study.

The risk score does not always increase with age in our nomogram. Previous research indicated that young cancer patients suffered a higher risk of lymph node metastasis 27-29, which may be the reason why the risk score of PDAC patients under 50 is higher than that of ones aged 51-60. Limitations of this study include: (1) the use of retrospective data; (2) detailed treatment information for included patients were not recorded in the SEER cohort, and we cannot investigate specific options, including R0 or not, preoperative or postoperative chemotherapy in the survival of PDAC patients; (3) other important factors, such as proximity/involvement of major vascular structures, CA 19-9, and patient comorbidities should also be minded.

## Conclusion

Our study confirmed that the recommendation suggesting all patients with PDAC after pancreatectomy receive chemotherapy is unreasonable. The novel risk-scoring system based on survival nomogram could serve as a chemotherapy decision tool and is capable of identifying low-risk patients with resected PDAC, who can avoid enduring side effects caused by chemotherapy without affecting the survival time.

## Supplementary Material

Supplementary figures.

## Figures and Tables

**Table 1:** Characteristics of patients with PDAC after surgery

Characteristics	Total (n=8691)		With chemotherapy(n=6346)				Without chemotherapy (n=2345)	
			Training group (n=4231)		Verification group (n=2115)			
	N	%	N	%	N	%	N	%
**Gender**								
Female	4266	49.09%	2051	48.48%	1047	49.50%	1168	49.81%
Male	4425	50.91%	2180	51.52%	1068	50.50%	1177	50.19%
**Age (years)**								
≤40	106	1.22%	49	1.16%	31	1.47%	26	1.11%
41-50	605	6.96%	318	7.52%	182	8.61%	105	4.48%
51-60	1976	22.74%	1081	25.55%	506	23.92%	389	16.59%
61-70	3020	34.75%	1557	36.80%	795	37.59%	668	28.49%
71-80	2366	27.22%	1042	24.63%	516	24.40%	808	34.46%
>80	618	7.11%	184	4.35%	85	4.02%	349	14.88%
**Marital status**								
Married	5527	63.59%	2809	66.39%	1394	65.91%	1324	56.46%
Unmarried/NOS	3164	36.41%	1422	33.61%	721	34.09%	1021	43.54%
**Race**								
White	7243	83.34%	3533	83.50%	1800	85.11%	1910	81.45%
Black	867	9.98%	431	10.19%	189	8.94%	247	10.53%
Other/NOS	581	6.69%	267	6.31%	126	5.96%	188	8.02%
**Tumor location**								
Head	6425	73.93%	3205	75.75%	1581	74.75%	1639	69.89%
Body/Tail	1380	15.88%	624	14.75%	339	16.03%	417	17.78%
Other	886	10.19%	402	9.50%	195	9.22%	289	12.32%
**Pathological grade**								
I	780	8.97%	358	8.46%	161	7.61%	261	11.13%
II	3857	44.38%	1852	43.77%	908	42.93%	1097	46.78%
III	2859	32.90%	1397	33.02%	729	34.47%	733	31.26%
IV	71	0.82%	40	0.95%	17	0.80%	14	0.60%
Unknown	1124	12.93%	584	13.80%	300	14.18%	240	10.23%
**T stage**								
T1a-b	201	2.31%	69	1.63%	34	1.61%	98	4.18%
T1c	1173	13.50%	517	12.22%	278	13.14%	378	16.12%
T2	4680	53.85%	2313	54.67%	1163	54.99%	1204	51.34%
T3	1858	21.38%	926	21.89%	459	21.70%	473	20.17%
T4	591	6.80%	324	7.66%	142	6.71%	125	5.33%
Tx	188	2.16%	82	1.94%	39	1.84%	67	2.86%
**N stage**								
N0	2975	34.23%	1357	32.07%	676	31.96%	942	40.17%
N1	3231	37.18%	1629	38.50%	813	38.44%	789	33.65%
N2	1864	21.45%	944	22.31%	479	22.65%	441	18.81%
Nx	621	7.15%	301	7.11%	147	6.95%	173	7.38%
**M stage**								
M0	8148	93.75%	3959	93.57%	1984	93.81%	2205	94.03%
M1	543	6.25%	272	6.43%	131	6.19%	140	5.97%
**Radiotherapy**								
Neoradiotherapy	581	6.69%	387	9.15%	184	8.70%	10	0.43%
Radiotherapy^A^	2861	32.92%	1797	42.47%	897	42.41%	167	7.12%
No	5249	60.40%	2047	48.38%	1034	48.89%	2168	92.45%
**RNE**								
≤5	1629	18.74%	751	17.75%	372	17.59%	506	21.58%
6-10	1633	18.79%	772	18.25%	363	17.16%	498	21.24%
11-15	1855	21.34%	914	21.60%	441	20.85%	500	21.32%
16-20	1452	16.71%	721	17.04%	390	18.44%	341	14.54%
>20	1997	22.98%	1014	23.97%	519	24.54%	464	19.79%
NOS	115	1.32%	49	1.16%	30	1.42%	36	1.54%
**Extension**								
Localized	1608	18.50%	684	16.17%	350	16.55%	574	24.48%
Extrapancreatic	7083	81.50%	3547	83.83%	1765	83.45%	1771	75.52%

RNE: Regional nodes examined; NOS: Not otherwise specified. A: not neoadjuvant.

**Table 2:** Univariable and multivariable Cox regression model analyses for nomogram

Characteristics	Univariable analysis				Multivariable analysis			
	OR	95% CI lower	95% CI upper	*p*-value	OR	95% CI lower	95% CI upper	*p*-value
**Gender**				0.022				0.018
Female		reference				reference		
Male	1.084	1.012	1.162	0.022	1.088	1.015	1.167	0.018
**Age (years)**				0.001				0.001
≤40		reference				reference		
41-50	0.959	0.684	1.346	0.811	1.057	0.753	1.485	0.748
51-60	0.919	0.667	1.268	0.607	0.982	0.711	1.356	0.913
61-70	0.980	0.712	1.348	0.899	1.070	0.777	1.473	0.680
71-80	1.117	0.810	1.540	0.500	1.221	0.884	1.685	0.226
>80	1.169	0.822	1.663	0.384	1.229	0.862	1.751	0.254
**Marital status**				0.235				
Married		reference				NA		
Unmarried/NOS	1.045	0.972	1.125	0.235				
**Race**				0.037				0.044
White		reference				reference		
Black	0.982	0.876	1.101	0.760	1.016	0.905	1.141	0.787
Other/NOS	0.821	0.706	0.955	0.010	0.826	0.709	0.962	0.014
**Tumor location**				.963				
Head		reference				NA		
Body/Tail	0.989	0.894	1.093	0.824				
Other	1.008	0.895	1.136	0.894				
**Pathological grade**				<0.001				<0.001
I		reference				reference		
II	1.267	1.106	1.451	0.001	1.229	1.070	1.410	0.003
III	1.704	1.484	1.957	<0.001	1.614	1.402	1.857	<0.001
IV	1.439	0.979	2.115	0.064	1.650	1.120	2.431	0.011
Unknown	1.233	1.051	1.446	0.010	1.118	0.947	1.319	0.187
**T stage**				<0.001				<0.001
T1a-b		reference				reference		
T1c	1.427	1.017	2.003	0.039	1.051	0.744	1.484	0.779
T2	1.814	1.309	2.512	<0.001	1.228	0.879	1.716	0.227
T3	2.176	1.564	3.028	<0.001	1.423	1.014	1.997	0.041
T4	2.540	1.799	3.587	<0.001	1.598	1.116	2.287	0.010
Tx	2.214	1.484	3.301	<0.001	1.385	0.920	2.087	0.119
**N stage**				<0.001				<0.001
N0		reference				reference		
N1	1.510	1.386	1.644	<0.001	1.471	1.345	1.608	<0.001
N2	1.941	1.762	2.139	<0.001	1.992	1.791	2.216	<0.001
Nx	1.927	1.672	2.221	<0.001	1.347	1.133	1.602	0.001
**M stage**				<0.001				<0.001
M0		reference				reference		
M1	2.057	1.805	2.343	<0.001	1.737	1.512	1.996	<0.001
**Radiotherapy**				0.014				0.109
Neoradiotherapy		reference				reference		
Radiotherapy^A^	1.079	0.947	1.230	0.254	0.950	0.826	1.093	0.474
No	1.172	1.029	1.334	0.017	1.029	0.896	1.182	0.683
**RNE**				<0.001				<0.001
≤5								
6-10	0.910	0.814	1.017	0.096	0.859	0.757	0.974	0.018
11-15	0.830	0.745	0.926	0.001	0.759	0.670	0.861	<0.001
16-20	0.785	0.699	0.881	<0.001	0.668	0.585	0.763	<0.001
>20	0.782	0.702	0.871	<0.001	0.629	0.553	0.715	<0.001
NOS	0.984	0.720	1.345	0.920	0.910	0.664	1.247	0.557
**Extension**				<0.001				<0.001
Localized		reference				reference		
Extrapancreatic	1.455	1.320	1.603	<0.001	1.259	1.136	1.395	<0.001

RNE: Regional nodes examined; NOS: Not otherwise specified, NA: Unavailable. A: not neoadjuvant.

**Table 3:** The risk score of each independent prognostic factor

Characteristics	Points
**Age (years)**	
≤40	3
41-50	11
51-60	0
61-70	13
71-80	33
>80	35
**Race**	
White	27
Black	30
Other/NOS	0
**Gender**	
Female	0
Male	12
**Pathological grade**	
I	0
II	30
III	70
IV	70
Unknown	18
**T stage**	
T1a-b	0
T1c	5
T2	28
T3	50
T4	66
Tx	46
**N stage**	
N0	0
N1	56
N2	100
Nx	45
**M stage**	
M0	0
M1	84
**RNE**	
≤5	67
6-10	45
11-15	28
16-20	9
>20	0
NOS	53
**Extension**	
Localized	0
Extrapancreatic	33
